# Optimizing Fodder Yield and Quality Through Grass–Legume Relay Intercropping in the Mediterranean Region

**DOI:** 10.3390/plants14060877

**Published:** 2025-03-11

**Authors:** Antigolena Folina, Panteleimon Stavropoulos, Antonios Mavroeidis, Ioannis Roussis, Ioanna Kakabouki, Eleni Tsiplakou, Dimitrios Bilalis

**Affiliations:** 1 Laboratory of Agronomy, Department of Crop Science, Agricultural University of Athens, 11855 Athens, Greece; stavropoulosp@aua.gr (P.S.); amauroeidis@aua.gr (A.M.); roussis@aua.gr (I.R.); i.kakabouki@aua.gr (I.K.); bilalis@aua.gr (D.B.); 2 Laboratory of Nutritional Physiology & Feeding, Department of Animal Science, Agricultural University of Athens, 11855 Athens, Greece; eltsiplakou@aua.gr

**Keywords:** intercropping, RCI, aggressivity, RYM, RY, relative leaf area index, ELER

## Abstract

An annual relay intercropping of grasses and legumes (LGI) (50:50) was compared with the sole crops, respectively, to determine the effect of the mixtures on the yield and quality of them as fodder in the Mediterranean region. The treatments were sole Rye (*Secale cereale*; G1), Ryegrass (*Lolium perenne;* G2), Faba bean (*Vicia faba* L.; L1), Berssem (*Trifolium alexandrinum* L.; L2), inoculated Clover (*Trifolium alexandrinum* L.; L3), and all the combinations of grasses and legumes. The experiment used a randomized block design with three blocks. ANOVA showed significant effects of intercropping on the biomass yield (BY) and the forage quality. Monocrops L2 and L3 showed better fodder quality than LGI and L1. The relative land-use efficiency (RLI) was higher for four out of six intercrops, while G2L1 and G2L3 had an RLI < 1, indicating lower efficiency than their monocrops. The Aggressivity Index (AG) showed that L1 was competitive against the grasses. The Relative Yield Maximization (RYM) demonstrated that intercropping significantly improved the biomass yield. The competition indices revealed that G1 with legumes had the highest efficiency and economic viability (ELER > 1), while the G2 combinations were less profitable. The study highlights the importance of selecting species based on soil fertility and climatic conditions to optimize intercropping outcomes.

## 1. Introduction

Intercropping, the agricultural practice of growing two or more crops simultaneously within the same field, is a sustainable strategy for optimizing resource utilization and improving overall productivity [1,2]. Intercropping enhances land-use efficiency, stabilizes yields, and improves soil health by combining species with complementary growth habits, nutrient requirements, or pest resistance, thereby optimizing the utilization of resources such as light, water, and nutrients [3,4,5]. This diversity often reduces the incidence of pests and diseases, as monoculture-associated vulnerabilities are minimized [3]. Furthermore, the legumes used in intercropping systems improve soil fertility by fixing atmospheric nitrogen, reducing the need for synthetic fertilizers [6]. Intercropping can also increase land-use efficiency, measured through the Land Equivalent Ratio (LER), often surpassing monocropping yields [7,8]. Additionally, diversified cropping systems enhance ecological stability, contribute to biodiversity conservation, and provide farmers with multiple sources of income and food security [9].

However, despite its advantages, intercropping presents several challenges that require careful consideration. Successful implementation demands meticulous planning and management, as improper crop selection, spacing, or species combinations can intensify the intra- and interspecific competition for essential resources such as light, water, and nutrients, potentially leading to reduced yields instead of the intended productivity gains [10]. Additionally, crop growth dynamics, root interactions, and allelopathic effects must be carefully balanced to avoid negative competitive interactions [11,12]. From an operational perspective, intercropping increases in complexity at every stage of cultivation. Planting, weeding, fertilization, and harvesting all become more labor-intensive and time-consuming, as different species may require varying inputs, growth periods, and management practices [13]. This can raise labor demands and production costs, making intercropping less attractive for large-scale farming operations that are reliant on streamlined processes [14]. Mechanization also poses significant challenges, as most modern agricultural machinery is designed for uniform, single-crop systems, making it difficult to efficiently sow, maintain, and harvest mixed crops [15]. Furthermore, the successful adoption of intercropping requires specialized agronomic knowledge. Farmers must understand crop compatibility, optimal planting densities, and nutrient cycling dynamics, which can serve as a barrier for those unfamiliar with the practice. A lack of access to scientific guidance, technical support, and localized research on suitable crop combinations further complicates widespread adoption [16].

Legume–grass intercropping (LGI) is a strategic agricultural practice that combines the complementary traits of legumes and grasses to maximize both yield and forage quality. In LGI systems, legumes contribute significantly to nitrogen enrichment in the soil through biological nitrogen fixation, reducing the dependency on synthetic fertilizers [17]. This nitrogen becomes available to grasses, promoting their growth and increasing the overall biomass production of the mixture. Grasses, on the other hand, excel at the efficient use of resources, particularly water and sunlight, and contribute to the stability of the system by suppressing weeds through their rapid growth and ground-covering ability. The interaction between legumes and grasses in LGI systems often results in a synergistic effect, where legumes enhance the protein content of the fodder, making it nutritionally superior for livestock, while grasses contribute to a higher dry matter yield [18]. This balance between quality and quantity is particularly valuable in forage systems [19]. Research has shown that LGI systems can improve land-use efficiency, measured through indices such as LER, and can increase resilience to environmental stresses, such as droughts or nutrient-poor soils [20,21,22]. Furthermore, the diverse root systems of legumes and grasses improve soil structure and health, contributing to long-term agricultural sustainability [23]. The need for LGI in forage and pasture systems is driven by the growing demand for sustainable, high-quality livestock feed and the challenges of maintaining soil health and productivity in diverse agroecosystems [24]. LGI offers a practical solution to these challenges by combining the complementary strengths of legumes and grasses [25].

This balance between quality and quantity is crucial for meeting the nutritional demands of livestock, particularly in intensive farming systems. Additionally, legumes have the unique ability to fix atmospheric nitrogen, reducing the need for chemical fertilizers, which can be costly and environmentally damaging. This natural nitrogen enrichment benefits the companion grass species, promoting their growth and overall system productivity [26,27]. Moreover, the diverse root systems of legumes and grasses improve soil structure, reduce erosion, and enhance water infiltration and retention, which is especially valuable in regions prone to droughts or poor soil conditions. LGI systems also provide ecological benefits by supporting biodiversity, reducing pest and disease pressure, and improving carbon sequestration in soils. These systems are particularly important in forage and pasture management in areas like the Mediterranean, where climatic variability and resource constraints demand efficient and resilient cropping strategies. By integrating LGI into forage systems, farmers can achieve a more sustainable balance of economic profitability, environmental stewardship, and livestock productivity, making it a vital practice for modern agriculture.

In recent years, Greece has experienced notable shifts in the consumption of animal products [28]. A study by the Research Institute of Retail Consumer Goods in 2023 reported a 16% decrease in the consumption of primary animal proteins, equating to approximately 6 kg less meat per person annually. Conversely, there has been a rise in the consumption of plant-based proteins, particularly legumes. Additionally, the same study highlighted a 42% reduction in dairy product consumption, notably in full-fat fresh milk, while low-fat milk and egg consumption remained stable or increased. The decreasing consumption of animal products is closely linked to growing concerns about the environmental impacts of livestock farming. Livestock production is a significant contributor to greenhouse gas emissions, deforestation, and excessive water usage [29]. To mitigate these environmental challenges, sustainable agricultural practices are gaining attention. Intercropping is emerging as an eco-friendly approach to cultivating animal feed [2]. By adopting intercropping strategies for animal feed production, it is possible to reduce the environmental footprint of livestock farming. This approach not only supports sustainable agriculture but also addresses consumer concerns regarding the ecological impacts of animal product consumption. At the same time, the increasing global demand for animal products derived from organic systems highlights the need for sustainable and environmentally friendly agricultural practices [30]. Organic systems prioritize natural inputs and ecological balance, making practices like LGI essential. LGI reduces the reliance on synthetic fertilizers, as legumes naturally fix atmospheric nitrogen, enriching the soil for companion grasses and subsequent crops [31]. This not only lowers production costs but also minimizes the environmental footprint associated with fertilizer manufacturing and use, such as greenhouse gas emissions and water pollution. Moreover, the complementary growth patterns of legumes and grasses optimize resource use, improving soil health, enhancing biodiversity, and fostering resilient ecosystems [32]. By integrating LGI into forage systems, farmers can support organic livestock production, offering high-quality animal feed while adhering to the principles of sustainability and environmental stewardship [33]. This approach aligns with consumer preferences for organic, ethically produced animal products and contributes to a more sustainable agricultural future [34].

The primary objective of this study was to evaluate the effects of various relay LGI combinations (Rye, Ryegrass, Faba bean, Berseem, and inoculated Clover) on biomass yield and fodder quality under the challenging conditions of Mediterranean semi-arid environments. We tested six distinct legume–grass mixtures to determine which combination offered the highest productivity and best nutritional quality, as assessed through a carefully designed experimental framework. In addition to evaluating yield and quality, we used four competition indices to further analyze the interactions between the crops, allowing us to identify the most efficient and sustainable mixture. Furthermore, an economic analysis was conducted to compare the profitability of the different intercrop combinations, providing a comprehensive assessment of both agronomic performance and financial viability. This research aims to provide valuable insights into optimizing forage systems for both high biomass production and improved feed quality, while considering the competitive dynamics between legumes and cereals in intercropping systems.

## 2. Results

### 2.1. ANOVA Analysis and Hsu’s Multiple Comparisons

**Figure 1:**
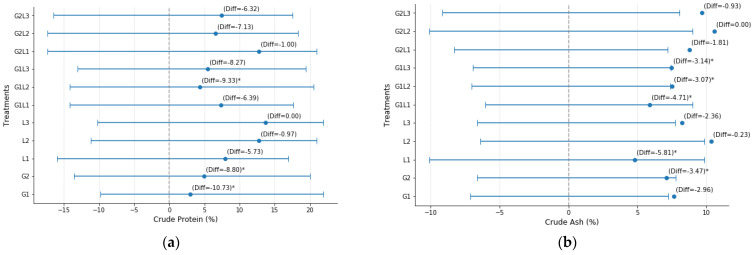

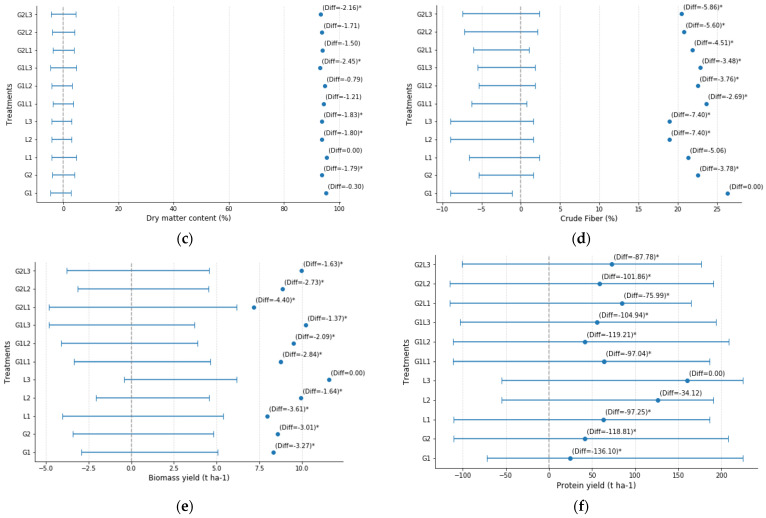
Hsu’s Multiple Comparisons with the Best (MCB) for (**a**) Crude Protein (CP) (**b**) Crude Ash (CA) (**c**) Dry Matter Content (DMC) (**d**) Crude Fiber (CF) (**e**) Biomass Yield (BY) and (**f**) Protein Yield (PY) across different monocropping and intercropping treatments. The values are presented along with their 95% confidence intervals (CI), including lower and upper bounds. Negative difference values indicate the deviation from the highest-performing treatment, with asterisks (*) denoting statistically significant differences at *p* < 0.05.

According to Table 1, the ANOVA results indicate that the intercropping treatments had a statistically significant impact on multiple measured traits, whereas the block effect was not explicitly tested. The analysis highlights notable differences among the treatments, particularly for CP (F = 2.66, *p* < 0.05), suggesting that cropping combinations played a crucial role in determining forage protein levels. The statistical analysis using Hsu’s MCB test for Crude Protein (CP) highlights distinct differences across the evaluated treatments (Figure 1a). The mean CP varied from 2.967% (G1) to 13.7% (L3). Several treatments, including G1, G1L2, G1L3, and G2, exhibited significantly lower protein percentages compared to the highest-yielding treatment, as indicated by the negative differences with statistical significance (*p* < 0.05). Among the monocrops, L3 achieved the highest CP (13.7%), setting it apart from the other treatments. In contrast, G1 (−10.733), G1L2 (−9.333), and G2 (−8.800) recorded the lowest values, highlighting the substantial reductions in CP. Certain intercropping combinations, such as G2L2 (−7.133) and G1L3 (−8.267), also exhibited moderate declines compared to the best-performing treatment. The results indicate that L3 outperformed L2 in several key agronomic and quality parameters, particularly in CP. The higher CP content in L3 suggests that inoculation contributed to enhanced nitrogen fixation, leading to improved protein synthesis in the plant tissue.

Similarly, CA (F = 4.85, *p* < 0.01) exhibited highly significant variation, indicating that the different intercropping treatments influenced the mineral composition of the forage (Table 1). According to Figure 1, the results of Hsu’s Multiple Comparisons with the Best (MCB) test for CA indicate significant variations among treatments. The mean CA values ranged from 4.787% (L1) to 10.593% (G2L2). Treatments G1L1, G1L2, G1L3, G2, and L1 exhibited significantly lower CA content compared to the best-performing treatments, as indicated by the negative differences with statistical significance (*p* < 0.05) (Figure 1b). Among the monocrops, L1 had the lowest CA (−5.807, *p* = 0.000), differing significantly from the other treatments. In contrast, L2 (10.367%) and G2L2 (10.593%) had the highest CA values, with differences not statistically significant compared to the best treatment. Intercropping combinations such as G1L1 (−4.707), G1L2 (−3.063), and G1L3 (−3.137) showed a notable reduction in CA compared to the highest values observed in some of the monocrop and intercrop treatments.

The effect on DMC (F = 2.69, *p* < 0.05), although less pronounced, still suggests a treatment-dependent trend in moisture retention and forage preservation (Table 1). As shown in Figure 1c, Hsu’s Multiple Comparisons with the Best (MCB) test confirms notable disparities in DMC among the treatments. The mean DMC values ranged from 93.05% (G1L3) to 95.50% (L1). Treatments G1L3 (93.05%), G2 (93.71%), G2L3 (93.34%), L2 (93.70%), and L3 (93.67%) exhibited significantly lower DMC compared to the best-performing treatments, as indicated by the negative differences with statistical significance (*p* < 0.05). Among the monocrops, L1 (95.502%) had the highest DMC, showing no significant difference from the best-performing group. Conversely, intercropping combinations such as G1L3 (−2.452), G2 (−1.792), and G2L3 (−2.154) had significantly lower DMC, suggesting that the inclusion of certain grass–legume combinations may result in reduced DMC.

More strikingly, CF (F = 13.17, *p* < 0.001) showed a highly significant effect, emphasizing that the species interactions within intercropping systems strongly influenced the structural composition of the plants (Table 1). According to the results of Hsu’s Multiple Comparisons with the Best (MCB) test for CF, significant variations were observed among the different treatments. The mean CF values ranged from 18.937% (L2 and L3) to 26.34% (G1). Several treatments, including G2, L1, L2, and L3, exhibited significantly lower CF content compared to the best-performing treatment, as indicated by the negative differences with statistical significance (*p* < 0.05) (Figure 1d). Among the monocrops, L2 and L3 recorded the lowest CF (−7.403), differing significantly from the other treatments. In contrast, G1 (26.34%) had the highest CF content, with a statistically significant difference compared to the other treatments. Intercropping combinations such as G1L1 (−2.687), G1L2 (−3.76), and G1L3 (−3.483) also showed notable reductions in CF compared to the highest observed values.

The most substantial differences through ANOVA analysis were observed in BY (F = 13.78, *p* < 0.001), confirming that intercropping significantly affected overall productivity and PY (F = 4.86, *p* < 0.01), reinforcing the role of species combinations in optimizing protein output per hectare (Table 1). The mean BY ranged from 7.173 t ha^−1^ (G2L1) to 11.571 t ha^−1^ (L3). Several treatments, including G1, G1L1, G1L2, G1L3, G2, G2L1, G2L2, G2L3, and L1, exhibited significantly lower BY compared to the highest-yielding treatment, as indicated by their negative differences with statistical significance (*p* < 0.05). Among the monocrops, L3 recorded the highest biomass yield (11.571 t ha^−1^), making it the best-performing treatment. In contrast, G2L1 (−4.397) and L1 (−3.607) exhibited the lowest yields, with notable reductions in biomass production. Similarly, several intercropping combinations, such as G1L1 (−2.841) and G2L2 (−2.727), showed a significant decline in biomass accumulation compared to L3 (Figure 1e). Additionally, L3 achieved the highest biomass yield, reinforcing that biological nitrogen fixation played a crucial role in supporting plant growth and overall productivity.

Based on the MCB test results in Figure 1, significant differences in PY were observed among the treatments (Figure 1f). PY ranged from 24.61 t ha^−1^ (G1) to 160.71 t ha^−1^ (L3). Treatments G1, G1L1, G1L2, G1L3, G2, G2L1, G2L2, G2L3, and L1 showed significantly lower yields compared to the highest-yielding L3 (*p* < 0.05). The lowest yields were seen in G1 (−136.10), G1L2 (−119.21), and G2 (−118.81). Intercropping combinations like G2L3 (−87.78) and G1L3 (−104.95) also had markedly lower yields than L3.

### 2.2. Impact of Intercropping on Forage/Feed Digestibility

**Figure 2:**
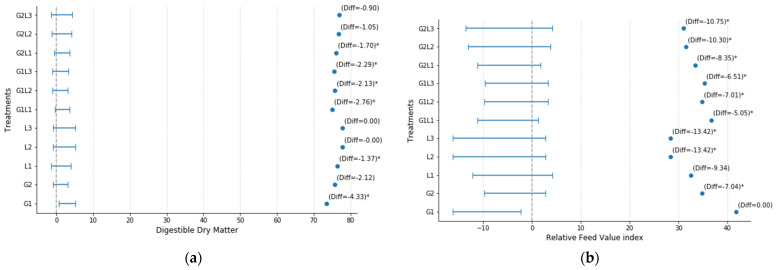
Hsu’s Multiple Comparisons with the Best (MCB) for (**a**) Digestible Dry Matter (DDM), and (**b**) Relative Feed Value (RFV) across different monocropping and intercropping treatments. The values are presented along with their 95% confidence intervals (CI), including lower and upper bounds. Negative difference values indicate the deviation from the highest-performing treatment, with asterisks (*) denoting statistically significant differences at *p* < 0.05.

According to the results of Hsu’s Multiple Comparisons with the Best (MCB) test for DDM, significant variations were observed among treatments (Figure 2a). The mean DDM values ranged from 73.51% (G1) to 77.84% (L3). Several treatments, including G1 (−4.33), G1L1 (−2.76), G1L2 (−2.13), G1L3 (−2.293), and G2L1 (−1.697), exhibited significantly lower DDM content compared to the highest-performing treatment (L3). Among the monocrops, L3 recorded the highest DDM value (77.84%), while G1 had the lowest, with a significant difference. Intercropping treatments such as G2L2 (−1.053) and G2L3 (−0.903) showed smaller differences from the highest-performing treatments and were not statistically significant.

The Hsu’s Multiple Comparisons with the Best (MCB) test for Relative Feed Value (RFV) revealed significant variations among treatments. The mean RFV values ranged from 28.39 (L3) to 41.807 (G1). Several treatments, including G2 (−7.043), L2 (−13.423), L3 (−13.417), G1L1 (−5.053), G1L2 (−7.01), G1L3 (−6.51), G2L1 (−8.35), G2L2 (−10.297), and G2L3 (−10.753), exhibited significantly lower RFV than the highest-performing treatment (G1). Among the monocrops, G1 had the highest RFV, whereas L2 and L3 had the lowest, with notable reductions in the forage quality. Intercropping treatments such as G1L2 and G2L2 also showed substantial declines compared to the best treatment.

### 2.3. Advanced Competition Indices

**Figure 3:**
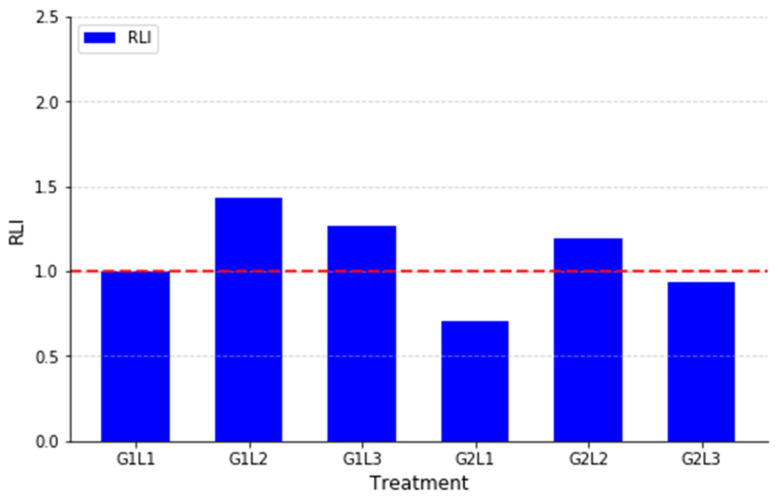
Relative Leaf Area Index (RLI) for intercropping treatments. The red line at 1.0 represents the threshold where intercropping and monocropping have equal leaf area efficiency.

The RLI index was calculated to evaluate the leaf area efficiency in the intercropping systems compared to the monocrops. The results indicate that most intercropping treatments improved LAI efficiency, particularly in G1L2 (1.43) and G1L3 (1.27), which exhibited significantly higher RLI values, suggesting that these combinations enhanced canopy development and light interception compared to their respective monocrops (Figure 3).

Similarly, G2L2 (1.19) showed a moderate increase in LAI, reinforcing the potential complementary interactions between G2 and L2. In contrast, G2L1 (0.71) and G2L3 (0.94) demonstrated RLI values below 1, indicating that these intercropping systems did not improve LAI efficiency compared to their monocrops.

Interestingly, G1L1 (1.00) had an RLI of exactly 1, meaning that the intercropping combination did not significantly alter LAI efficiency compared to its individual monocrops.

#### 2.3.1. Indicators for Quantifying the Intensity of Competition

**Figure 4:**
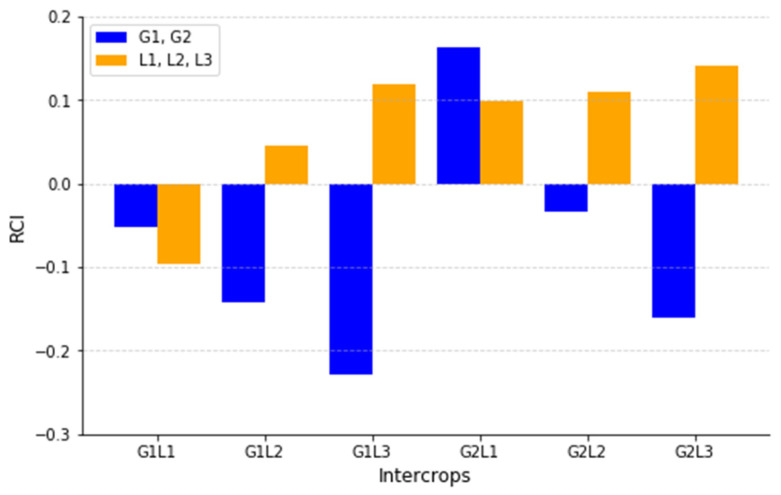
Relative Competition Intensity (RCI) for intercropping treatments.

According to Figure 4, the RCI index was calculated to assess the competitive interactions between the grasses and legumes in the intercropping systems. The results reveal significant differences in the competition intensity among the treatments, indicating which species faced suppression or benefited from intercropping.

Across the intercropping combinations, G1L3 (−0.229, 0.118) and G2L3 (−0.161, 0.141) exhibited the highest negative RCI values for grasses, suggesting that the grasses in these systems faced competitive pressure from the legumes. In contrast, G2L1 (0.162, 0.099) and G2L2 (−0.033, 0.110) displayed positive RCI values for grasses, indicating that the grasses in these mixtures maintained a competitive advantage over the legumes.

Interestingly, G1L1 (−0.052, −0.097) showed a relatively balanced competition, suggesting the mild suppression of both species in this combination. This indicates that Rye (G1) and Bean (L1) coexisted with moderate competition stress, making this mixture more stable compared to the strongly competitive interactions in the other intercropping treatments. Based on the RCI index, it is evident that the intensity of competition varies depending on the intercropping combination. Some species, particularly Bean (L1), Clover (L2), and inoculated Clover (L3), were more likely to suppress the grasses in certain treatments, whereas the grasses in the other systems (e.g., G2L1) maintained dominance over the legumes.

According to Table 2, the AG index was used to assess the competitive interactions between the grasses (G1, G2) and legumes (L1, L2, L3) in the intercropping systems. The results indicate which species exerted a stronger competitive influence, highlighting the dominance relationships between the grasses and legumes. The highest positive A(G) values, indicating strong dominance of grasses over legumes, were observed in G1L3 (0.695, −0.695) and G2L3 (0.604, −0.604). This suggests that Rye (G1) and Lolium (G2) were significantly more competitive than inoculated Clover (L3) in their respective combinations, suppressing the legume’s growth. Similarly, G1L2 (0.375, −0.375) and G2L2 (0.285, −0.285) also exhibited positive A(G) values, indicating that the grasses maintained a competitive advantage over Clover (L2), although to a lesser extent compared to inoculated Clover (L3).

Conversely, negative AG (G) values were recorded in G1L1 (−0.090, 0.090) and G2L1 (−0.126, 0.126). These results suggest that Bean (L1) displayed a slight competitive advantage over both Rye (G1) and Lolium (G2), although the competition was relatively balanced in these mixtures compared to the others. However, the findings from the AG index showed that the grasses tended to dominate the intercropping mixtures, particularly in the combinations with inoculated Clover (L3) and Clover (L2).

The RCI values indicated balanced competition between the grasses and legumes in the G1L1 combination, suggesting the mild suppression of both species. However, the AG index suggested that the legumes exerted greater competitive pressure over the grasses. While these results may seem contradictory, they reflect the different aspects of interspecies competition. The RCI measures the overall balance of competition, while the AG index highlights the direction of competitive influence. The observed discrepancy may be due to differences in how each index quantifies competitive interactions, with AG emphasizing resource dominance by one species over another.

#### 2.3.2. Indicators for Quantifying the Effects of Competition

**Figure 5:**
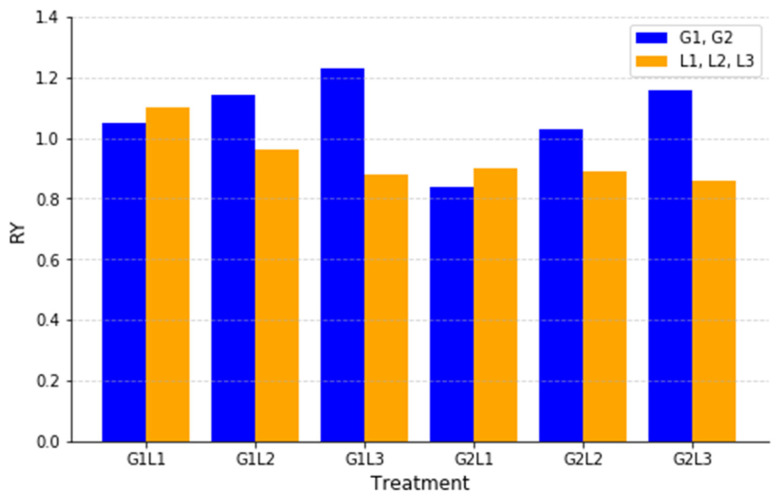
Relative Yield (RY) for intercropping treatments.

Figure 5 illustrates that the RY index was calculated to assess the effects of competition in the intercropping systems by comparing the yield of each species in the intercropping system relative to its monocrop yield. This index helped determine which species benefited from intercropping and which experienced competitive suppression.

In general, the grasses (G1, G2) exhibited higher RY values than the legumes in most treatments, indicating that the grasses performed better in intercropping than in monocropping. The highest RY(G) values were observed in G1L3 (1.23) and G2L3 (1.16), suggesting that Rye (G1) and Lolium (G2) had the greatest competitive advantage when intercropped with inoculated Clover (L3). Similarly, G1L2 (1.14) and G2L2 (1.03) showed positive RY values for grasses, indicating that Clover (L2) did not strongly suppress their growth.

Conversely, the legume RY values were generally lower than those of the grasses, suggesting that legumes experienced more competitive pressure in intercropping systems. The lowest RY(L) values were found in G1L3 (0.88) and G2L3 (0.86), meaning that inoculated Clover (L3) was the most suppressed legume in these combinations. However, in G1L1 (1.10) and G2L1 (0.90), Bean (L1) performed relatively well, indicating that this species was more competitive and less affected by intercropping than other legumes. The RY index clearly indicates that grasses tend to dominate in intercropping systems, while legumes, particularly inoculated Clover (L3), are more susceptible to competition.

**Figure 6:**
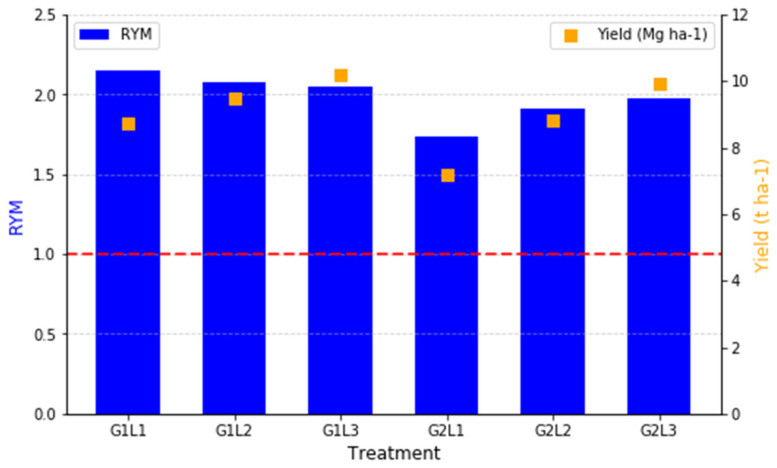
Relative Yield Monoculture (RYM) and Biomass Yield (BY) for intercropping treatments. The red line at 1.0 represents the threshold where intercropping and monocropping perform equally, with values above 1.0 indicating a yield advantage for intercropping.

According to Figure 6, the RYM index was calculated to assess the combined productivity of the intercropping systems compared to the monocultures. Unlike RY, which evaluates species-specific competition effects, RYM provides an overall measure of whether the intercropping system as a whole is more or less productive than the average monocrop performance. RYM values of greater than one indicate that intercropping is more productive than monocropping, while values of lower than one would suggest that the system is less efficient. In all the intercropping treatments, the RYM values exceeded one, confirming that each intercropping combination resulted in higher total productivity than monocropping. The highest RYM values were observed in G1L1 (2.148), G1L2 (2.080), and G1L3 (2.053), suggesting that Rye (G1) intercropped with Bean (L1), Clover (L2), and inoculated Clover (L3) led to the most efficient land use and resource allocation. Similarly, G2L3 (1.975) and G2L2 (1.912) also exhibited notably high RYM values, demonstrating that Lolium (G2) was also effective in intercropping, although slightly less than Rye (G1).

Among the treatments, G2L1 (1.736) had the lowest RYM value, indicating that Lolium (G2) intercropped with Bean (L1) was the least efficient combination in terms of total productivity. However, since all the RYM values remained above one, this still indicates a net advantage of intercropping over monocropping for all the tested combinations. The RYM results reinforce the benefits of intercropping, particularly when pairing Rye (G1) with legumes.

### 2.4. Economic Analysis of Intercropping vs. Monocropping

**Figure 7:**
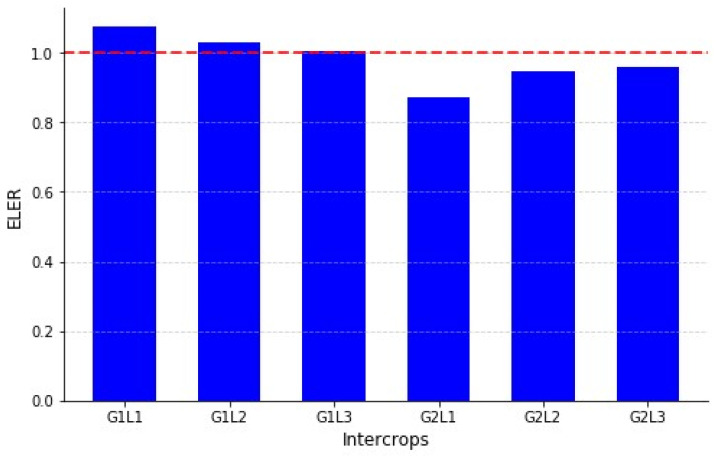
Economic Land Equivalent Ratio (ELER) for intercropping treatments. The red line at 1.0 represents the threshold where intercropping and monocropping have equal economic efficiency, with values above 1.0 indicating a financial advantage for intercropping.

The ELER was calculated for all the intercropping combinations to assess their economic viability compared to the monoculture systems. Figure 7 presents the ELER values for the different intercropping combinations. The highest ELER value was observed in the G1L1 combination (1.076), indicating that intercropping Grass 1 (G1) with Legume 1 (L1) resulted in the most profitable system. Other intercropping combinations with ELER values greater than one, such as G1L2 (1.029) and G1L3 (1.006), also demonstrated economic benefits over monoculture. Conversely, the intercropping combinations G2L1 (0.872), G2L2 (0.948), and G2L3 (0.970) yielded ELER values below one, suggesting that monoculture was a more profitable approach for these specific combinations.

The ELER values indicate that the intercropping systems involving G1 tended to be more economically advantageous compared to those involving G2. The G2L3 combination, with an ELER of 0.970, approached the economic efficiency threshold but did not surpass it, reinforcing that monoculture remains a more viable option in this case.

## 3. Discussion

Intercropping grasses with forage legumes is a widely recognized approach that enhances forage production and quality by improving silage yield and protein content compared to monocultures [35,36,37]. Legumes generally produce protein-rich forage, while grasses are more fibrous, creating a complementary balance when intercropped [38]. This synergy improves forage nutritional quality, contributing to a more balanced diet for livestock [18]. Numerous studies support this, including the one by Barsila et al. (2024), which found that vetch monocultures and a 50:50 oat/vetch seed rate proportion resulted in a higher CP than pure oat monocultures [39]. Similarly, intercropping maize with legumes increased crude protein yield and dry matter yield while reducing fiber fractions, improving digestibility [40,41]. Our findings align with these results, as intercropping positively influenced forage quality by balancing protein and fiber content. Legumes consistently maintained the highest crude protein levels, which contributed to improved overall forage quality. One possible mechanism behind these results is the complementary resource use between grasses and legumes, where legumes fix atmospheric nitrogen, benefiting the associated grasses and enhancing overall system productivity. Additionally, reduced competition for specific soil nutrients in mixed cropping systems may further explain the improved protein content and digestibility. These outcomes highlight the potential of legume–grass intercropping to reduce the need for synthetic nitrogen fertilizers through biological nitrogen fixation, promoting more sustainable agricultural practices [25].

The CA content was found to vary significantly among forage treatments, with 100% oats and the 75:25 oat/vetch mix showing the highest values [39]. Our results indicate that legume inclusion, especially in the G1-based mixtures and L1 monocrop, lowered CA content, potentially affecting mineral composition and digestibility. In contrast, G2L2 and L2 maintained higher CA levels, influencing forage quality. Similar trends were observed in alfalfa-based systems [42] and Napier grass intercropped with cowpea, where CA increased from 13.05% to 16.00% between cuts [43].

The previous studies have shown that DMC varies depending on intercropping combinations. Bacchi et al. (2021) found that ryegrass intercropped with vetch had a DMC of 309.2 g/kg, while ryegrass intercropped with clover exhibited a slightly higher DMC of 319.7 g/kg compared to 322.9 g/kg in the ryegrass monoculture [44]. Similarly, research on cereal–legume intercrops indicated that barley intercropped with vetch had a DMC of 348.6 g/kg, while triticale–vetch intercrops reached 358.4 g/kg, both significantly higher than the legume monocultures, which ranged from 152.1 g/kg (pea) to 178.3 g/kg (clover) [44]. In line with these insights, our results show that while the L1 monocrop maintained the highest dry matter content, intercropping systems such as G1L3, G2L3, L2, and L3 resulted in a significant reduction, potentially affecting forage preservation and digestibility.

Research has consistently demonstrated that intercropping plays a crucial role in modifying CF composition by reducing Neutral Detergent Fiber (NDF) and Acid Detergent Fiber (ADF) concentrations, thereby enhancing forage digestibility [40,45,46,47]. Additionally, intercropping corn with legumes not only decreased fiber content but also enhanced microbial activity in the root zone, potentially improving nutrient availability and forage breakdown [47]. In line with these outcomes, our results indicate that certain intercropping systems and specific monocrops, particularly L2 and G2L2, significantly reduced the fiber content, which may enhance forage digestibility and feeding efficiency. Conversely, G1 maintained the highest fiber concentration, which is undesirable as excessive CF reduces digestibility and lowers the nutritional value of forage. This result is particularly significant given that, as forage matures, CP becomes diluted while fiber content increases, making the forage less digestible. Since CF represents the indigestible portion of the forage, its levels should be minimized to optimize feed quality. Legumes help to counter-act this issue by providing a more digestible and protein-rich forage component, which is crucial for balancing animal diets. Indeed, legumes generally produce higher-quality forage than grasses, as noted by many researchers [48].

BY is a crucial determinant of productivity in LGI systems, with numerous studies demonstrating that certain mixtures outperform the monocultures [49]. Guinea grass–centro mixtures at ratios of 2:2 and 3:1 showed superior performance compared to their respective monocultures [50], while berseem clover–barley intercropping enhanced both the forage and protein yields [51]. Similarly, faba bean–rye intercrops yielded the highest forage and crude protein outputs [15]. In Mediterranean environments, LGI at a 50:50 ratio provided yield advantages of 16.0% for fodder and 11.5% for protein relative to the monocultures [44]. These increases are primarily attributed to complementary ecological interactions, such as nitrogen fixation by legumes, which enhances nutrient availability for grass species [52]. Contrary to these observations, our results show that L3 (inoculated clover monocrop) was the most productive treatment in terms of biomass yield. In contrast, the mixtures with G1 all had a higher yield compared to its monoculture. This could be attributed to competitive interactions between species, including resource competition, which may limit the growth and biomass accumulation of certain species within the mixture. Additionally, species-specific growth limitations or suboptimal environmental conditions could further restrict productivity in these systems.

PY is also a crucial determinant of productivity in LGI systems, with numerous studies demonstrating that certain intercrops outperform monocultures [53]. Legumes demonstrated the highest protein yield, significantly contributing to the enhancement of overall protein yield in intercrop systems, thereby improving the nutritional quality of the mixed forage. On the other hand, Guinea grass–centro mixtures at ratios of 2:2 and 3:1 exhibited superior performance compared to their respective monocultures [50], while berseem clover–barley intercropping enhanced both forage and protein yields [51]. Similarly, faba bean–rye intercrops produced the highest forage and crude protein outputs [15].

Significant differences in DDM and RFV were observed among the forage treatments, highlighting the contrasting nutritional benefits of grasses and legumes. Legumes (L3, L2) exhibited superior digestibility but lower feed value, while grasses (G1) had higher RFV but lower digestibility. The results correspond with the previous research, including that of Muttappanavar and Shekara, who recorded a DDM of 63.71% and an RFV of 119.2 in a B × N hybrid + Desmanthus intercropping system, as well as that of Prajapati et al., who reported higher DDM (67.3%) and RFV (126.8%) in sweet sorghum–cowpea intercropping [54,55]. The improved digestibility in legume–grass systems can be attributed to reduced fiber content, enhanced nitrogen efficiency, and improved light interception, all of which enhance forage intake and livestock nutrition [56,57]. The study suggests that strategic intercropping could help reduce reliance on feed supplements by providing nutrient-rich forage, contributing to more sustainable livestock systems. However, limitations exist, as only the crude fiber was measured, and the seasonal variations in the forage composition were not considered. Future research should include detailed fiber fractionation (ADF, NDF, lignin content), feeding trials to validate livestock performance, and long-term studies on forage quality stability under different environmental conditions.

The RLI index, which evaluates leaf area efficiency, revealed that G1L2 (1.43) and G1L3 (1.27) had significantly higher values, indicating improved canopy development and light interception. In contrast, G2L1 and G2L3 exhibited lower RLI values, suggesting that these combinations were less effective, likely due to competition for light or space, which limited LAI efficiency. Optimizing planting density in maize–soybean strip intercropping further improved total LAI and grain yield [8], emphasizing the role of species compatibility and planting density in enhancing the light capture and overall productivity in intercropping systems.

The AG index measures the competitive advantage of one forage species over another in intercropping, with positive values indicating dominance. In our study, faba beans imposed strong competition on grasses, whereas two-leaf clovers did not. Similarly, in Sudan grass–vetch intercropping, Sudan grass was significantly (*p* < 0.01) more aggressive than vetch in most treatments, except at a 25:75 (S:V) seed rate, where vetch dominated (−0.71). At a 50:50 ratio, Sudan grass exhibited higher AG (3.68), likely due to inter-species competition dynamics [58]. Research suggests that legumes tend to be more competitive at lower grass–legume ratios, while grasses dominate when their proportion increases [50,59]. This aligns with our findings, where faba beans successfully competed against grasses, whereas two-leaf clovers were less competitive. Comparable trends have been observed in forage sorghum–legume intercropping systems, where sorghum was the dominant crop, while cowpea was the most competitive legume [60]. In contrast, barley–faba bean (100:62.5) intercropping showed a balanced competition, with barley having an AG value of −0.20 and faba bean 0.20 [61]. Similarly, Zhang et al. (2011) reported an AG value of 0.19 for alfalfa relative to corn [62]. In the Guinea grass–*Centrosema pubescens* mixtures, the legume was dominant at a 1:3 ratio, while Guinea grass became more aggressive at the ratios of 2:2 and 3:1 [50]. Likewise, in maize–palisade grass–pigeon pea intercropping, maize was dominant, while the grass and legume components had low AG values, indicating minimal competition [63].

The RYM index assesses the productivity of intercropping compared to monocropping, with values above one indicating a yield advantage [64]. In our study, all the mixtures achieved RYM > 1, demonstrating the superiority of intercropping over monocropping. Among the legumes, Faba bean (L1) intercropped with Ryegrass (G2) had the lowest intercropping advantage. Garnier et al. (1997) observed RYM values ranging from 0.87 to 1.19 in a substitutive legume–grass intercropping design and 1.15 to 2.82 in an additive design, emphasizing the impact of experimental approaches on intercropping outcomes [65]. Similarly, Wilson (1988) found that legume–grass systems typically achieved RYM values between 1.1 and 2.5, further supporting the yield benefits of intercropping despite the potential competitive interactions [66].

The RCI index reveals varying competition intensities across the intercropping treatments in our study. Grasses (G1, G2) experienced the strongest suppression in G1L3 and G2L3, indicating significant competitive pressure from inoculated Clover (L3). Conversely, the grasses maintained dominance in G2L1 and G2L2, while the G1L1 combination showed balanced competition, suggesting a moderate coexistence between Rye (G1) and Bean (L1). These results highlight the critical role of species selection in managing competition and optimizing intercropping efficiency. In line with these outcomes, the previous studies have shown that RCI is useful for assessing the competition dynamics in intercropping systems, with species’ performance compared to monocrops [67,68]. A previous study on legume–grass mixtures, such as Berseem clover and ryegrass, found that increasing the legume proportion led to higher dry forage yields, but the RCI values indicated no significant advantage over monocultures [53].

RY is a key metric for assessing the competition in intercropping systems, with higher values indicating species’ dominance. Our study revealed that the grasses (G1, G2) consistently outperformed the legumes, suggesting a competitive advantage in mixed cropping. Rye (G1) and Ryegrass (G2) thrived best with inoculated Clover (L3), while Clover (L2) had minimal suppressive effects. In contrast, the legumes faced greater competition, with inoculated Clover (L3) being the most suppressed. However, Faba bean (L1) showed resilience, particularly in G1L1 (1.10), suggesting its competitive adaptability. These findings align with the research demonstrating that grasses typically exhibit higher RY values than legumes, reinforcing their dominance in intercropping systems [50]. Studies on intermediate wheatgrass–legume mixtures also reported increasing RY values over time, highlighting the long-term advantages of LGI [69]. Similarly, *Medicago ruthenica* intercropped with *Bromus inermis* led to significant forage yield increases, emphasizing the role of species complementarity in productivity [70]. The AG and RCI values showed that L3 was more susceptible to suppression in certain intercropping combinations, particularly when paired with highly competitive grasses such as Rye (G1) and Lolium (G2). This suggests that while inoculated clover offers improved productivity and forage quality, its ability to compete for resources may be slightly weaker compared to classic clover in mixed cropping systems.

The study’s findings reveal that the intercropping combinations involving G1 and the various legume species yielded ELER values of greater than one, indicating superior economic efficiency over the monoculture systems. In contrast, the combinations with G2 and legumes resulted in ELER values of lesser than one, suggesting that the monoculture is more economically viable for these specific pairings. This lower economic efficiency for G2 may be attributed to differences in crop growth characteristics and nutrient demands. Lolium exhibits a high nutrient requirement and a competitive growth habit, which may limit the productivity of the legume component in intercropping systems. Additionally, the slow initial growth of legumes could have further reduced overall biomass accumulation and economic returns when combined with Lolium. These results align with those of the other researchers, who demonstrated that cassava-based intercropping systems in Nigeria achieved higher net revenues per unit of land compared to sole cropping, with ELER values ranging from 1.00 in the cassava monoculture to 1.94 in the cassava–yam–maize–vegetable combinations [71]. Similarly, Koocheki et al. (2021) reported that saffron-based intercropping systems increased ELER values, with the highest ELER values recorded under optimal irrigation conditions, supporting the idea that resource efficiency influences economic performance [72]. The insights suggest that intercropping can improve economic efficiency by optimizing land use and increasing profitability, although benefits vary by species combination. However, the study is limited by specific environmental conditions, the lack of detailed cost analysis, and the exclusion of long-term market fluctuations. Future research should explore long-term economic trends, optimize planting densities, and assess policy incentives for sustainable intercropping.

## 4. Materials and Methods

### 4.1. Experimental Design

The experiment followed a Randomized Complete Block Design (RCBD) with three replicates (blocks) to control environmental variability, including soil heterogeneity, microclimatic differences, and field position effects. This design helps to minimize the impact of factors such as soil texture variation, moisture distribution, and nutrient availability across the experimental field, ensuring that differences in biomass yield and fodder quality are primarily due to the treatments rather than external environmental influences.

**Figure 8:**
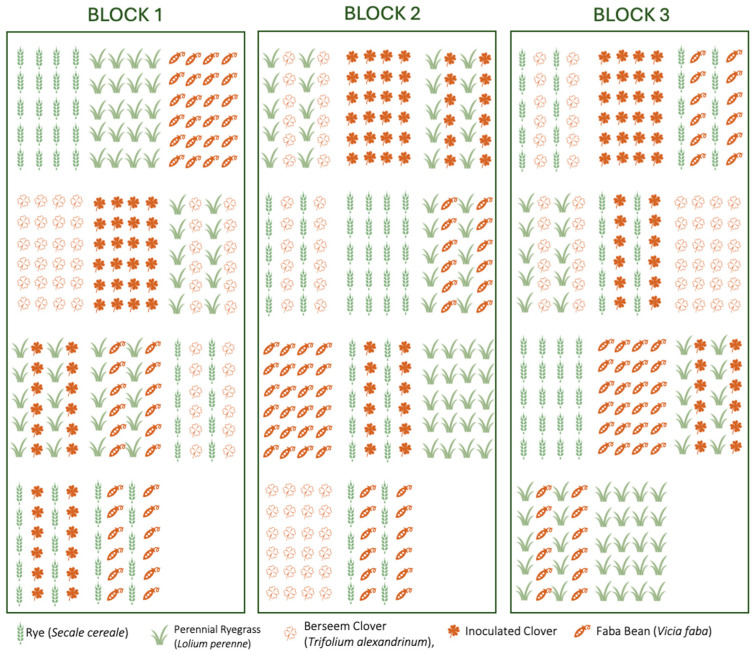
The experimental design.

The experimental area covered 175.5 m^2^, divided into 3 blocks/replicates, each containing 11 plots of 4.5 m^2^ (Figure 8). Soil tillage included primary tillage with an agronomic chisel at a depth of 20 cm, followed by secondary tillage with a disc harrow. The plants were sown in November using a 10-row manual seed planter. A sprinkler irrigation system was applied over the soil surface, providing water only during the first three weeks to support early crop establishment.

The treatments consisted of five monocrops (Rye (G1), Lolium (G2), Bean (L1), Broad Clover (L2), and inoculated Clover (L3)) and six intercropping combinations (Rye + Bean (G1L1), Rye + Broad Clover (G1L2), Rye + inoculated Clover (L1G3), Lolium + Bean (G1L1), Lolium + Broad Clover ((L2G2), and Lolium + inoculated Clover (L2G3)), making a total of 11 treatments. Randomization within blocks was conducted by assigning treatments randomly within each block to minimize spatial variability and ensure unbiased results. The plots were designed at 4 m × 5 m (20 m^2^), with a row spacing of 20 cm and a 50 cm gap between the plots to prevent competition effects outside the designed intercropping systems. A seeding ratio of 50% cereal and 50% legume was used, meaning that the total seeding rate in intercropping was equally divided between the two crop types on an area basis.

### 4.2. Experimental Site and Environmental Conditions

The experimental site for the intercropping study was located at the Agricultural University of Athens, on the organic field of the Laboratory of Agronomy (coordinates 37°59′01.83″ N, 23°42′07.37″ E, altitude 30 m). The study was conducted during the 2016–2017 growing period, evaluating both intercrops and monocrops.

The soil texture at the experimental site was classified as clay loam (CL), composed of 35.9% sand, 29.8% clay, and 34.3% silt. The soil pH was 7.29 (measured in a 1:1 water H_2_O solution), and the organic matter content was 2.37% (determined using the Wakley and Black method, 1934) [73].

**Figure 9:**
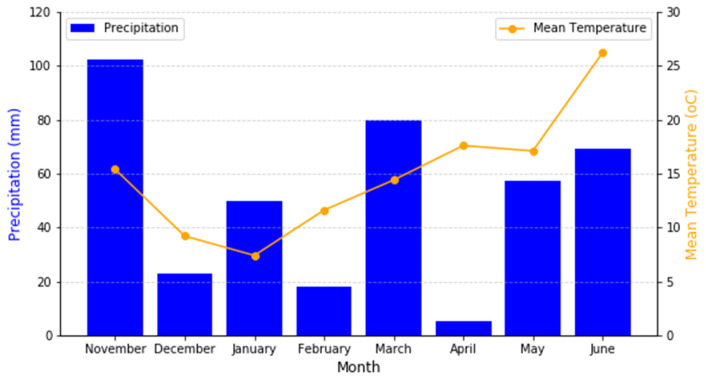
Climate data.

The climatic conditions of the site, including precipitation and average temperature, are presented in Figure 9. The graph illustrates the monthly mean temperature (°C) and total precipitation (mm) recorded between November 2016 and May 2017, which correspond to the crop-growing period. The total precipitation for this period was 317.7 mm. These climatic factors are critical for understanding the environmental conditions under which the intercropping and monocropping systems were evaluated.

### 4.3. Plant Material

The study utilized five different plant species commonly used in Mediterranean forage systems, selected for their agronomic benefits and complementarity in intercropping. The species included Rye (*Secale cereale*), Perennial Ryegrass (*Lolium perenne*), Berseem Clover (*Trifolium alexandrinum*), inoculated Clover (*Trifolium alexandrinum* inoculated with nitrogen-fixing bacteria), and Faba Bean (*Vicia faba*). These species were chosen based on their nutritional value, adaptability to semi-arid conditions, and their ability to enhance soil fertility in sustainable forage production systems.

Grasses such as Rye and Perennial Ryegrass were selected due to their high biomass productivity, competitive growth, and ability to improve soil structure. The legumes, including Berseem Clover and Faba Bean, were incorporated for their nitrogen-fixing ability, which enhances soil fertility, reducing the need for synthetic fertilizers. The inoculated Clover was specifically used to evaluate the impact of biological nitrogen fixation in improving overall forage quality. The inoculated Clover was inoculated with *Rhizobium trifolii*, a bacterial strain commonly used for nitrogen fixation in legume crops. The inoculation process ensured that the clover formed an effective symbiotic relationship with the rhizobia, enhancing biological nitrogen fixation and improving plant growth without the need for synthetic fertilizers.

### 4.4. Crop Management and Agronomic Practices

The field experiment was established in November 2016, with sowing conducted using a 10-row manual seed planter to ensure uniform seed distribution. The seed rates for each species were as follows: Rye (Secale cereale) at 1.7 kg ha^−1^, Perennial Ryegrass (*Lolium perenne*) at 11 kg ha^−1^, Berseem Clover (*Trifolium alexandrinum*) at 3.5 kg ha^−1^, inoculated Clover (*Trifolium alexandrinum*) at 3.5 kg ha^−1^, and Faba Bean (*Vicia faba*) at 3.0 kg ha^−1^. The row spacing was set to 20 cm to optimize plant growth. The plant-to-plant distance varied depending on the crop species, following the standard agronomic practices for each. In intercropping, similar plant-to-plant spacing was maintained within each crop type (grass crops and legume crops) to ensure uniform growth and fair comparison. No additional fertilization was applied throughout the entire growing period. Similarly, no irrigation was provided beyond the initial establishment phase, as the experiment relied solely on natural precipitation. Weed management was conducted manually, with weeds removed by hand to avoid chemical interference with the cropping system. No significant pest or disease outbreaks were observed during the experiment, eliminating the need for additional control measures. Harvest was conducted 178 days after sowing (DAS), with biomass yield assessed using a 0.25 m^2^ quadrat, collecting five sample per treatment to ensure representative data.

### 4.5. Data Collection

#### 4.5.1. Forage Quality Analysis and Digestibility Indices

For proximate composition analysis, the plant samples were collected 109 days after sowing (DAS), corresponding to the fodder stage. The collected samples were ground to pass through a 1 mm Wiley mill screen (Thomas T4274.E15 Steel Model 4 Wiley Mill; Arthur H. Thomas, Philadelphia, PA, USA) to ensure uniformity in sample preparation. The processed samples were then analyzed using a fully automated Kjeldahl analyzer (Kjeltec 8400; Foss Tecator AB, Höganas, Sweden) to determine their chemical composition. The quality parameters assessed included Crude Ash (CA, %) (AOAC 924.05), Dry Matter Content (DMC, %) (AOAC 943.01), Crude Fat (CF, %) (AOAC 920.39), Crude Fiber (CF, %) (AOAC 978.10), and Crude Protein (CP, %), which was calculated by multiplying the nitrogen concentration by 6.25, following the standard AOAC conversion factors [74,75].

To evaluate forage quality, Digestible Dry Matter (DDM) and Relative Feed Value (RFV) were calculated based on fiber content analysis. DDM represented the proportion of dry matter that was digestible by ruminants and was inversely related to the fiber content, particularly Acid Detergent Fiber (ADF) [76,77]. It can be determined using the following equation:

(1) DDM=88.9−(0.779∗ADF)

where ADF (%) is expressed as a percentage of dry matter. Higher ADF values indicate lower digestibility, as the indigestible fiber components increase.

The Relative Feed Value (RFV) index was calculated to assess forage quality and livestock intake potential, integrating both Neutral Detergent Fiber (NDF) and DDM. RFV provides a standardized measure of forage quality relative to a baseline forage standard [78]. The equation used was as follows:

(2) $$RFV=(\frac{120}{NDF})*\left(\frac{DDM}{100}\right)*100$$

where NDF (%) represents the total fiber fraction affecting voluntary intake, while DDM (%) estimates digestibility. A higher RFV indicates better forage quality, with greater digestibility and intake potential. Forages with RFV values above 100 are considered to have high quality, supporting better animal performance and feed efficiency [77]. These indices are widely applied in forage evaluation to compare different cropping systems and optimize feed formulations in livestock production.

#### 4.5.2. Biomass Yield (BY) and Protein Yield (PY)

Biomass Yield was determined at harvest using a 0.25 m^2^ quadrat, with one quadrat collected per treatment to ensure representativeness at 178 DAS. Within each plot, five subsamples were randomly selected, and their biomass values were averaged for statistical analysis. The collected plant material was then weighed directly in its dry form to determine the final biomass yield.

The Protein Yield (PY) was calculated using the following equation:

(3) $$PY=\sum_{i=1}^{n}\left(Protein\;Contenti*DMi\;\right)$$

where n = 11, representing the different intercropping and monocropping treatments. This calculation provided an estimate of the total protein output per unit area by integrating the crude protein percentage with the corresponding dry matter (DM) yield of each treatment. The resulting values allowed for a direct comparison of protein productivity across treatments, highlighting the impact of intercropping on forage quality [79].

#### 4.5.3. Competition Indices

The plant competition was calculated according to the table below (Table 3). Competition intensity in the intercropping systems was assessed using indices that measured how strongly one species suppressed another. These indices helped distinguish whether species interactions resulted in competition, neutrality, or facilitation. The Relative Competition Index (RCI) evaluated the competitive suppression a species experienced in an intercrop compared to its monoculture, while the Aggressivity Index (AG) determined which species was more dominant within the system. Both indices provided insights into the competitive balance and species dominance in mixed cropping environments. Beyond measuring the competition intensity, certain indices assessed the outcomes of species interactions in the intercropping systems. The Relative Yield Monoculture (RYM) and Relative Yield (RY) indices compared the productivity of a species in the intercrop versus monoculture settings. These indices indicated whether intercropping led to complementary resource use, yield advantages, or competitive disadvantages, helping to assess overall system efficiency.

#### 4.5.4. Relative LAI Index (RLI)

The Relative Leaf Area Index (RLI) was used to assess the efficiency of intercropping in terms of canopy development compared to monocropping systems. Adapted from the Land Equivalent Ratio (LER), this index quantified whether intercropping enhanced or reduced the leaf area development relative to the monocultures. The RLI was calculated using the following formula:

(4) $$RLI=\frac{{LAI}_{intercrop}}{{(LAI}_{mono1}+{LAI}_{monno2})/2}$$

where LAI_intercrop represents the Leaf Area Index (LAI) of the intercropping system, while LAI_mono1 and LAI_mono2 correspond to the LAI values of the respective monocrops.

An RLI value greater than 1 indicates that intercropping leads to greater canopy development, suggesting enhanced light interception and potential productivity advantages. An RLI of 1 signifies no difference between intercropping and monocropping, while an RLI below 1 suggests that monocropping results in a higher LAI, likely due to reduced competition within single species stands. By comparing LAI across cropping systems, the RLI provides insights into how interspecific interactions influence canopy structure, biomass accumulation, and overall resource use efficiency.

#### 4.5.5. Economic Analysis

To evaluate the economic efficiency of intercropping compared to monoculture, the Economic Land Equivalent Ratio (ELER) was calculated following the methodology adapted from economic land-use efficiency principles. The ELER provided a comparative measure of economic returns by incorporating both biomass yield and market value, ensuring a comprehensive assessment of cropping system profitability. The ELER was determined using the following formula:

(5) $$ELER=\frac{\left({Yield}_{intercrop,1}*{Price}_{crop,1}\right)+({Yield}_{intercrop,2}*{Price}_{crop,2})}{\left({Yield}_{mono,1}*{Price}_{crop,1}\right)+({Yield}_{mono,2}*{Price}_{crop,2})}$$

where:

- Yield_intercrop,1_, and Yield_intercrop,2_ represent the biomass yield (kg/ha) of grass forage and legume forage under intercropping conditions, respectively.
- Price_crop,1_ and Price_crop,2_ correspond to the market price ($/kg) of grass forage and legume forage, respectively.
- Yield_mono,1_ and Yield_mono,2_ denote the biomass yields of grass forage and legume forage when grown as sole crops (monoculture).

The market prices for the grass forage and the legume forage were obtained from the United States Department of Agriculture (USDA) Agricultural Prices Report (National Agricultural Statistics Service [NASS], 2022) [82]. According to this source, the price of grass forage was $164 per ton, and the price of legume forage was $209 per ton (NASS, USDA, 2022). These values were used to compute the economic returns for the intercropping and monoculture systems.

The ELER quantifies the relative economic advantage of intercropping. An ELER greater than 1 indicates that intercropping generates a higher economic return per unit land area than monoculture, suggesting improved land-use efficiency and profitability. Conversely, an ELER less than 1 implies that monoculture is more economically viable under the given conditions.

### 4.6. Statistical Analysis

The analysis of variance (ANOVA) was conducted using Statistica software 12 (StatSoft, Inc., Tulsa, OK, USA), employing a RCBD to assess the significance of differences among the treatments. ANOVA was chosen due to its ability to partition variance among multiple sources, allowing for the evaluation of the effects of intercropping on key agronomic traits while accounting for experimental variability. This statistical approach enabled the identification of significant interactions between the species in the intercropping systems, ensuring robust and reliable comparisons across treatments.

Hsu’s Multiple Comparisons with the Best (MCB) test was used to identify the most effective treatments by comparing each treatment against the best-performing one. Unlike standard pairwise comparisons, MCB specifically tests whether a treatment is significantly different from the best-performing group, providing a more targeted approach to ranking treatments. This method helps determine which intercropping or monocropping systems yield the highest biomass and protein while controlling for statistical errors. Analyses were conducted at a 95% confidence level, ensuring robust differentiation between treatments.

## 5. Conclusions

Legumes improved the quality of the mixtures, whereas grasses did not significantly increase yields, reinforcing the agronomic and economic advantages of legume–grass intercropping (LGI). The study demonstrates that species combinations significantly influence forage quality, productivity, and competition dynamics. Intercropping enhanced forage yield, crude protein content, and digestibility while reducing fiber concentrations, supporting its role in sustainable fodder production. Additionally, the results indicate that biological nitrogen fixation in legume–grass mixtures can reduce reliance on synthetic fertilizers, contributing to improved environmental sustainability.

Economic analysis showed that rye (G1) intercropped with legumes resulted in an ELER value greater than one, indicating superior efficiency over monoculture systems. In contrast, Ryegrass (G2) combinations were less economically viable. However, the economic feasibility of intercropping varies based on species selection, soil fertility, and climatic conditions, emphasizing the need for site-specific crop management strategies tailored to regional environmental factors.

A crucial aspect of successful intercropping is the careful selection of species and varieties based on the soil and climate conditions of each region. The adaptability of different forage crops to environmental factors such as precipitation, temperature, and soil nutrient availability plays a significant role in determining intercropping success. Future research should focus on optimizing species selection for different agroecological zones, refining planting densities, and exploring policy incentives to promote sustainable intercropping systems. Additionally, long-term studies on economic viability and environmental benefits will further strengthen the case for intercropping as a sustainable agricultural practice.

## Figures and Tables

**Table 1:** Analysis of Variance (ANOVA) for the Effects of Intercropping on Quality Parameters (Crude Protein (CP), Crude Ash (CA), Dry Matter Content (DMC), Crude Fiber (CF)), Forage Yield (Biomass Yield (BY), and Protein Yield (PY) and Digestibility Indices (Digestible Dry Matter (DDM), and Relative Feed Value (RFV)).

Source	DF	CP	CA	DMC	CF	BY	PY	DDM	RFV
**F_Blocks_**	2								
**F_Intercrop_**	10	2.66 *	4.85 **	2.69 *	13.17 ***	13.78 ***	4.86 **	13.17 ***	13.26 ***
**Error**	20								
**Total**	32								

F-test ratios are from ANOVA. Significance levels: * *p* < 0.05; ** *p* < 0.01; *** *p* < 0.001; ns, not significant (*p* > 0.05).

**Table 2:** Aggressivity Index (AG) values for grasses (G) and legumes (L) in different intercropping treatments.

Intercrops	AG (G)	AG (L)
G1L1	−0.090	0.09
G1L2	0.375	−0.375
G1L3	0.695	−0.695
G2L1	−0.126	0.126
G2L2	0.285	−0.285
G2L3	0.604	−0.604

**Table 3:** Indices for quantifying the intensity and effect of competition in intercropping systems.

Index	Formula	Limits			Introduced by
		Competition	Neutral	Facilitation	
Indices to quantify the intensity of competition					
Relative Competition Intensity	$RCI=\frac{Y_{monocrop\;}-Y_{intercrop}}{Y_{monocrop}}$	1	0	→ −∞	[67]
Aggressivity Index	$AG=\left(\frac{Y_{intercrop1}}{Y_{monocrop1}}\right)-\left(\frac{Y_{intercrop2}}{Y_{monocrop2}}\right)$	1	0	→ −∞	[80]
Indices to quantify the effect of competition					
Relative Yield Monoculture	$RYM=\frac{Y_{intercrop1}+Y_{intercrop2}}{\left(\frac{Y_{monocrop1}+Y_{monocrop2}}{2}\right)}$	<+1	1	>+1	[66]
Relative Yield	$RY=\frac{Y_{intercrop}}{Y_{monocrop}}$	<+1	1	>+1	[81]

## Data Availability

Data are contained within the article.

## Abbreviations

The following abbreviations are used in this manuscript:

CP	Crude Protein
CA	Crude Ash
CF	Crude Fiber
DMC	Dry Matter Content
BY	Biomass Yield
PY	Protein Yield
NDF	Neutral Detergent Fiber
ADF	Acid Detergent Fiber
DDM	Digestible Dry Matter
RFV	Relative Feed Value
RLI	Relative Leaf Area Index
ELER	Economic Land Equivalent Ratio
RCI	Relative Competition Intensity
AG	Aggressivity Index
RYM	Relative Yield Monoculture
RY	Relative Yield
MCB	Multiple Comparisons with the Best
ANOVA	Analysis of Variance
RCBD	Randomized Complete Block Design

## References

[1] Andrews D., Kassam A. (1976). The importance of multiple cropping in increasing world food supplies. Mult. Crop..

[2] Maitra S., Hossain A., Brestic M., Skalicky M., Ondrisik P., Gitari H., Brahmachari K., Shankar T., Bhadra P., Palai J.B. (2021). Intercropping—A low input agricultural strategy for food and environmental security. Agronomy.

[3] Huss C., Holmes K., Blubaugh C. (2022). Benefits and risks of intercropping for crop resilience and pest management. J. Econ. Entomol..

[4] Moreira B., Gonçalves A., Pinto L., Prieto Lage M.A., Carocho M., Caleja C., Barros L. (2024). Intercropping systems: An opportunity for environment conservation within nut production. Agriculture.

[5] Yin W., Chai Q., Zhao C., Yu A., Fan Z., Hu F., Fan H., Guo Y., Coulter J.A. (2020). Water utilization in intercropping: A review. Agric. Water Manag..

[6] Dwivedi A., Dev I., Kumar V., Yadav R.S., Yadav M., Gupta D., Singh A., Tomar S. (2015). Potential role of maize-legume intercropping systems to improve soil fertility status under smallholder farming systems for sustainable agriculture in India. Int. J. Life Sci. Biotechnol. Pharma Res..

[7] Bilalis D.J., Sidiras N., Kakampouki I., Efthimiadou A., Papatheohari Y., Thomopoulos P. (2005). Effects of organic fertilization on maize/legume intercrop in a clay loam soil and Mediterranean climate-Can the Land Equivalent Ratio (LER) index be used for root development?. J. Food Agric. Environ..

[8] Chen G., Jiang F., Zhang S., Zhang Q., Jiang G., Gao B., Cao G., Islam M.U., Cao Z., Zhao X. (2025). Potential crop yield gains under intensive soybean/maize intercropping in China. Plant Soil.

[9] Sanderson M.A., Archer D., Hendrickson J., Kronberg S., Liebig M., Nichols K., Schmer M., Tanaka D., Aguilar J. (2013). Diversification and ecosystem services for conservation agriculture: Outcomes from pastures and integrated crop–livestock systems. Renew. Agric. Food Syst..

[10] Freckleton R., Watkinson A. (1999). The mis-measurement of plant competition. Funct. Ecol..

[11] Himanen S.J., Mäkinen H., Rimhanen K., Savikko R. (2016). Engaging farmers in climate change adaptation planning: Assessing intercropping as a means to support farm adaptive capacity. Agriculture.

[12] Homulle Z., George T.S., Karley A.J. (2022). Root traits with team benefits: Understanding belowground interactions in intercropping systems. Plant Soil.

[13] Bilalis D.J., Karamanos A.J. (2010). Organic maize growth and mycorrhizal root colonization response to tillage and organic fertilization. J. Sustain. Agric..

[14] Chimonyo V.G.P., Modi A.T., Mabhaudhi T. (2015). Perspective on crop modelling in the management of intercropping systems. Arch. Agron. Soil Sci..

[15] Lithourgidis A., Dordas C. (2010). Forage yield, growth rate, and nitrogen uptake of faba bean intercrops with wheat, barley, and rye in three seeding ratios. Crop Sci..

[16] Mamine F., Farès M.h. (2020). Barriers and levers to developing wheat–pea intercropping in Europe: A review. Sustainability.

[17] Iannetta P.P., Young M., Bachinger J., Bergkvist G., Doltra J., Lopez-Bellido R.J., Monti M., Pappa V.A., Reckling M., Topp C.F. (2016). A comparative nitrogen balance and productivity analysis of legume and non-legume supported cropping systems: The potential role of biological nitrogen fixation. Front. Plant Sci..

[18] Kebede G., Assefa G., Feyissa F., Mengistu A. (2016). Forage legumes in crop-livestock mixed farming systems: A review. Int. J. Livest. Res..

[19] Eskandari H., Ghanbari A., Javanmard A. (2009). Intercropping of cereals and legumes for forage production. Not. Sci. Biol..

[20] Hamzei J., Seyedi M. (2015). Evaluation of the effects of intercropping systems on yield performance, land equivalent ratio, and weed control efficiency. Agric. Res..

[21] Belel M., Halim R., Rafii M., Saud H. (2014). Intercropping of corn with some selected legumes for improved forage production: A review. J. Agric. Sci..

[22] Xu Z., Li C., Zhang C., Yu Y., van der Werf W., Zhang F. (2020). Intercropping maize and soybean increases efficiency of land and fertilizer nitrogen use; A meta-analysis. Field Crops Res..

[23] Meena R.S., Das A., Yadav G.S., Lal R. (2018). Legumes for Soil Health and Sustainable Management.

[24] Bilalis D., Roussis I., Kakabouki I. (2024). Enhancing Crop Yield and Adaptability through Sustainable Soil Management: Effective and Eco-Friendly Practices. Plants.

[25] Gulwa U., Mgujulwa N., Beyene S.T. (2018). Benefits of grass-legume inter-cropping in livestock systems. Afr. J. Agric. Res..

[26] Rai S. (2024). Improved Agronomic Practices on Enhancement of Quality Forage Production for Livestock Farming. Agron. J. Nepal.

[27] Souza M.d.S., da Silva T.G.F., de Souza L.S.B., Jardim A.M.d.R.F., Araújo Júnior G.d.N., Alves H.M.N. (2019). Practices for the improvement of the agricultural resilience of the forage production in semiarid environment: A review. Amaz. J. Plant Res..

[28] Φωλίνα Ε.A. (2018). Aξιολόγηση δύο ειδών συγκαλλιέργειας σε συστήματα βιολογικής παραγωγής. Master’s Thesis.

[29] Gerber P.J., Steinfeld H., Henderson B., Mottet A., Opio C., Dijkman J., Falcucci A., Tempio G. (2013). Tackling Climate Change Through Livestock: A Global Assessment of Emissions and Mitigation Opportunities.

[30] Gamage A., Gangahagedara R., Gamage J., Jayasinghe N., Kodikara N., Suraweera P., Merah O. (2023). Role of organic farming for achieving sustainability in agriculture. Farming Syst..

[31] Wei Z., Maxwell T.M., Robinson B., Dickinson N. (2022). Legume nutrition is improved by neighbouring grasses. Plant Soil.

[32] Li S. (2024). Intercropping perennial cereal and legumes for improving biological soil health and microbial drought resilience. Acta Univ. Agric. Sueciae.

[33] Teshome A., Habte E., Teressa A., Muktar M.S., Assefa Y., Jones C.S. (2023). Methods and Practices for the Evaluation of Forage Legumes, Grasses and Fodder Trees for Use as Livestock Feeds.

[34] Sánchez-Bravo P., Chambers E., Noguera-Artiaga L., Sendra E., Chambers E., Carbonell-Barrachina Á.A. (2021). Consumer understanding of sustainability concept in agricultural products. Food Qual. Prefer..

[35] Papanaoum G., Bouloumpasi E., Lazaridou T.B. (2020). Silage yield and protein content of foragelegumes intercropping with cereals. AGROFOR.

[36] Gennatos K., Lazaridou T.B. (2021). Silage Yield and protein content of forage legumes intercropping with cereals in two spatial arrangements. AGROFOR.

[37] Zhang J., Yin B., Xie Y., Li J., Yang Z., Zhang G. (2015). Legume-cereal intercropping improves forage yield, quality and degradability. PLoS ONE.

[38] Vadez V., Krishnamurthy L., Kashiwagi J., Kholova J., Devi J., Sharma K., Bhatnagar-Mathur P., Hoisington D., Hash C., Bidinger F. (2007). Exploiting the functionality of root systems for dry, saline, and nutrient deficient environments in a changing climate. J. SAT Agric. Res..

[39] Barsila S.R., Acharya S., Acharya P. (2024). Herbage Mass Productivity, Composition, and Biological Compatibility of Oat and Vetch Mixture at Different Seed Rate Proportions in Abandoned Lands. Int. J. Agron..

[40] Javanmard A., Nasab A.D.M., Javanshir A., Moghaddam M., Janmohammadi H. (2009). Forage yield and quality in intercropping of maize with different legumes as double-cropped. J. Food Agric. Environ..

[41] Bilalis D., Papastylianou P., Konstantas A., Patsiali S., Karkanis A., Efthimiadou A. (2010). Weed-suppressive effects of maize–legume intercropping in organic farming. Int. J. Pest Manag..

[42] Xu R., Zhao H., You Y., Wu R., Liu G., Sun Z., Bademuqiqige, Zhang Y. (2022). Effects of intercropping, nitrogen fertilization and corn plant density on yield, crude protein accumulation and ensiling characteristics of silage corn interseeded into alfalfa stand. Agriculture.

[43] Suhailfayaz K., Singh R. (2023). The effect of Cowpea intercropping and different fertilizer levels on Nutritional quality of Napier grass. Ecol. Environ. Conserv..

[44] Bacchi M., Monti M., Calvi A., Lo Presti E., Pellicanò A., Preiti G. (2021). Forage potential of cereal/legume intercrops: Agronomic performances, yield, quality forage and LER in two harvesting times in a Mediterranean environment. Agronomy.

[45] Eskandari H. (2012). Yield and quality of forage produced in intercropping of maize (Zea mays) with cowpea (Vigna sinensis) and mungbean (Vigna radiate) as double cropped. J. Basic Appl. Sci. Res..

[46] Darko U., Svečnjak Z., Dujmović-Purgar D., Jareš D., Horvatić I. (2019). Influence of intercropping maize with climbing bean on forage yield and quality. Agrofor.

[47] Zaeem M., Nadeem M., Pham T.H., Ashiq W., Ali W., Gillani S.S.M., Moise E., Elavarthi S., Kavanagh V., Cheema M. (2021). Corn-soybean intercropping improved the nutritional quality of forage cultivated on podzols in boreal climate. Plants.

[48] Ball D., Collins M., Lacefield G., Martin N., Mertens D., Olson K., Putnam D., Undersander D., Wolf M. (2001). Understanding Forage Quality–American Farm Bureau Federation Publication 1 (01).

[49] Tahir M., Li C., Zeng T., Xin Y., Chen C., Javed H.H., Yang W., Yan Y. (2022). Mixture composition influenced the biomass yield and nutritional quality of legume–grass pastures. Agronomy.

[50] Baba M., Halim R., Alimon A., Abubakar I. (2011). Grass-legume mixtures for enhanced forage production: Analysis of dry matter yield and competition indices. Afr. J. Agric. Res..

[51] Vasilakoglou I., Dhima K. (2008). Forage yield and competition indices of berseem clover intercropped with barley. Agron. J..

[52] Luo F., Liu W., Mi W., Ma X., Liu K., Ju Z., Li W. (2023). Legume-grass mixtures increase forage yield by improving soil quality in different ecological regions of the Qinghai-Tibet Plateau. Front. Plant Sci..

[53] Rady A.M.S. (2016). Competition indices of berseem clover, Italian ryegrass mixtures. Alex. J. Agric. Sci..

[54] Prajapati B., Prajapati J., Kumar K., Shrivastava A. (2019). Determination of the relationships between quality parameters and yields of fodder obtained from intercropping systems by correlation analysis. Pan.

[55] Muttappanavar R.K., Shekara B. (2023). Quality contents of fodder as influenced by different perennial fodder intercropping systems. Forage Res..

[56] Song Y., Lee S.-H., Rahman M.A., Lee K.-W. (2021). Evaluation of intercropping sorghum× sudangrass hybrid (Sorghum bicolor) with legume crops based on growth characteristics, forage productivity, and feed values at a summer paddy field. J. Korean Soc. Grassl. Forage Sci..

[57] Tramacere L.G., Antichi D., Mele M., Ragaglini G., Mantino A. (2024). Effects of intercropping on the herbage production of a binary grass-legume mixture (*Hedysarum coronarium* L. and *Lolium multiflorum* Lam.) under artificial shade in Mediterranean rainfed conditions. Agrofor. Syst..

[58] Temesgen T. (2022). Effect Of Seed Rate On Forage Yield, Morphological Characteristics, and Chemical Composition of Sudan Grass (Sorghum Sudanense) and Vetch (Vicia Dasycarpa) Intercropping Grown under Irrigation Condition in North Mecha District of Ethiopia. Ph.D. Thesis.

[59] Basaran U., Dogrusoz M.C., Gulumser E., Mut H. (2017). Hay yield and quality of intercropped sorghum-sudan grass hybrid and legumes with different seed ratio. Turk. J. Field Crops.

[60] Azraf-ul-Haq Ahmad R.A., Mahmood N., Nazir M. (2006). Competitive performance of associated forage crops grown in different forage sorghum-legume intercropping systems. Pak. J. Agric. Sci..

[61] Agegnehu G., Ghizaw A., Sinebo W. (2006). Yield performance and land-use efficiency of barley and faba bean mixed cropping in Ethiopian highlands. Eur. J. Agron..

[62] Zhang G., Yang Z., Dong S. (2011). Interspecific competitiveness affects the total biomass yield in an alfalfa and corn intercropping system. Field Crops Res..

[63] Pariz C.M., Costa N.R., Costa C., Crusciol C.A.C., de Castilhos A.M., Meirelles P.R.d.L., Calonego J.C., Andreotti M., Souza D.M.d., Cruz I.V. (2020). An innovative corn to silage-grass-legume intercropping system with oversown black oat and soybean to silage in succession for the improvement of nutrient cycling. Front. Sustain. Food Syst..

[64] Williams A.C., McCarthy B.C. (2001). A new index of interspecific competition for replacement and additive designs. Ecol. Res..

[65] Garnier E., Navas M.-L., Austin M.P., Lilley J.M., Gifford R.M. (1997). A problem for biodiversity-productivity studies: How to compare the productivity of multispecific plant mixtures to that of monocultures?. Acta Oecologica.

[66] Wilson J.B. (1988). Shoot competition and root competition. J. Appl. Ecol..

[67] Wilson S.D., Keddy P.A. (1986). Measuring diffuse competition along an environmental gradient: Results from a shoreline plant community. Am. Nat..

[68] Šidlauskaitė G., Toleikienė M., Kadžiulienė Ž. (2024). Comparison of Productivity and Quality of Three Perennial Ryegrass Cultivars and Their Mixture in Response to Nitrogen Fertilization and Grass-Legume Mixtures. Plants.

[69] Crews T.E., Kemp L., Bowden J.H., Murrell E.G. (2022). How the nitrogen economy of a perennial cereal-legume intercrop affects productivity: Can synchrony be achieved?. Front. Sustain. Food Syst..

[70] Wei K., Xiang H., Liu Y., Zhang X., Yu X. (2024). Mixed cropping of Medicago ruthenica-Bromus inermis exhibits higher yield and quality advantages in the Longxi loess plateau region of Northwest China. Front. Sustain. Food Syst..

[71] Christopher C.O. (2008). Comparative analysis of enterprise combination costs and returns in cassava-based food crop farming systems in Delta State, Nigeria. ARPN J. Agri. Biol. Sci.

[72] Koocheki A., Moghaddam P.R., Seyyedi S.M. (2019). Saffron-pumpkin/watermelon: A clean and sustainable strategy for increasing economic land equivalent ratio under limited irrigation. J. Clean. Prod..

[73] Wakley A., Black I.A. (1934). An examination of the Degtiareff methods for determining soil organic matter and a proposed modification of chromic acid titration method. Soil Sci..

[74] AOAC (1990). Official Methods of Analysis of AOAC International.

[75] Helrich K. (1990). Official Methods of Analysis of the Association of Official Analytical Chemists.

[76] Schroeder J.W. (1994). Interpreting Forage Analysis.

[77] Undersander D., Moore J.E., Schneider N. (2002). Relative forage quality. Focus Forage.

[78] Rohweder D., Barnes R., Jorgensen N. (1978). Proposed hay grading standards based on laboratory analyses for evaluating quality. J. Anim. Sci..

[79] Nichiporovich A., Stroganova L., Vlasova M. (1961). Fotosinteticheskaya Deyatel’nost’rasteniy v Posevakh [Photosynthetic Activity of Plants in Crops].

[80] McGilchrist C., Trenbath B. (1971). A revised analysis of plant competition experiments. Biometrics.

[81] Keddy P.A. (1990). Competitive hierarchies and centrifugal organization in plant communities. Perspectives on Plant Competition.

[82] Statistics A. (1998). National Agricultural Statistics Service.

